# miR21 deletion in osteocytes has direct and indirect effects on skeletal muscle in a sex-dimorphic manner in mice

**DOI:** 10.1186/s13293-022-00465-9

**Published:** 2022-10-01

**Authors:** Alyson L. Essex, Padmini Deosthale, Joshua R. Huot, Hannah M. Davis, Nicholas Momeni, Andrea Bonetto, Lilian I. Plotkin

**Affiliations:** 1 Department of Anatomy, Cell Biology and Physiology, Indiana University School of Medicine, Indianapolis, IN USA; 2 Roudebush Veterans Administration Medical Center, Indianapolis, IN USA; 3 Indiana Center for Musculoskeletal Health, Indiana University, Indianapolis, IN USA; 4 Department of Surgery, Indiana University School of Medicine, Indianapolis, IN USA; 5 Simon Comprehensive Cancer Center, Indianapolis, IN USA; 6 Present Address: Ely Lilly and Company, Indianapolis, IN USA; 7 Present Address: University of Colorado Anschutz Medical Campus and University of Colorado Comprehensive Cancer Center, Aurora, CO USA

## Abstract

**Background:**

Osteocytic microRNA21 (miR21) removal alters cytokine production and bone mass by modulating osteoclast and osteoblast differentiation and activity. Removing osteocytic miR21 increases osteoclast/osteoblast numbers and bone mass in male mice, whereas it decreases osteoclasts/osteoblasts without affecting bone mass in female mice. On the other hand, it leads to sex-independent increases in bone mechanical properties. Because changes in bone remodeling and strength affect skeletal muscle through bone–muscle crosstalk, we investigated whether osteocytic miR21 deletion influences skeletal muscle.

**Methods:**

miR21fl/fl mice and 8kbDMP1-Cre mice were mated to obtain miR21-deficient mice primarily in the osteocyte (OtmiR21Δ) and littermate controls (miR21fl/fl). Four-month-old male and female mice were analyzed. Body composition was examined by DXA/Piximus and gene expression was assessed by qPCR. Ex vivo cultures of long bones devoid of bone-marrow cells from male and female 4-month-old were maintained for 48 h. Conditioned media were collected and used for the C2C12 assays. Two-way ANOVA analyses were performed to determine the contributions of genotype and sex and their interaction to the effects of miR21 deficiency.

**Results:**

Lean body mass was increased only in female OtmiR21Δ mice, although miR21 levels in soleus muscle were similar in miR21fl/fl (0.05 ± 0.02) and OtmiR21Δ (0.09 ± 0.04) mice. Female, but not male, OtmiR21Δ mice exhibited increased soleus (42%) and gastrocnemius (21%) muscle weight compared to miR21fl/fl littermates. However, muscle strength and gastrocnemius muscle fiber cross-sectional area were unaltered for either sex. Kinase phosphorylation (phospho/total protein ratio) in soleus muscle, measured as a surrogate for kinase activity by means of multiplex analysis, was also selectively changed depending on the mouse sex. Thus, female OtmiR21Δ mice had higher T185/Y187-ERK1/2 but lower S473-Akt phosphorylation than miR21fl/fl controls, while male OtmiR21Δ mice had higher S473-Akt phosphorylation, suggesting sex-dimorphic shifts in anabolic vs. catabolic signaling. Consistently, levels of FOXO3 and MuRF-1, known to be regulated by Akt, were only increased in male OtmiR21Δ mice. Atrogin-1 mRNA levels were upregulated in female OtmiR21Δ mice, suggesting a potential shift in protein regulation. Sex-specific effects were also found by exposing myotube cultures to conditioned media from 48-h-cultured marrow-flushed bones. Thus 5-day differentiated C2C12 myotubes treated with conditioned media of female OtmiR21Δ mice exhibit 12% higher average diameter compared to cells exposed to miR21fl/fl bone conditioned media. Yet, conditioned media from male bones had no effect on myotube size.

**Conclusions:**

We present a novel aspect of bone–muscle crosstalk in which osteocyte-derived miR21 influences skeletal muscle size, but not strength, in female but not male mice; whereas, intracellular signaling alterations resulting from loss of miR21 seem to alter protein dynamics in a sex-dimorphic fashion.

**Supplementary Information:**

The online version contains supplementary material available at 10.1186/s13293-022-00465-9.

## Highlights

- Osteocytic microRNA21 (miR21) removal in OtmiR21Δ mice alters cytokine production and bone mass by modulating osteoclast and osteoblast differentiation and activity.
- Only female, but not male, OtmiR21Δ mice exhibit higher lean body mass and soleus and gastrocnemius muscle weight compared to miR21fl/fl littermates.
- Sex-dependent consequences of osteocytic miR21 deletion on skeletal muscle were also found at protein and gene expression level.
- We conclude that osteocyte-derived miR21 influences skeletal muscle size, but not strength, in female but not male mice; whereas, intracellular signaling alterations resulting from loss of miR21 seem to alter protein dynamics in a sex-dimorphic fashion.

**Supplementary Information:**

The online version contains supplementary material available at 10.1186/s13293-022-00465-9.

## Introduction

Osteoporosis, a bone loss disease characterized by a decrease in bone mineral density (BMD), is a growing age-related disease estimated to have a global prevalence of 21.7% among the aged population [1]. Of particular interest is the increase in fracture risk associated with osteoporosis-related bone fragility. Fractures present a large economic cost and are known to lead to an increased risk for mortality [2, 3]. Importantly, low skeletal muscle mass, termed sarcopenia, is a known contributing factor for fall and fracture risk among aged patients [4, 5]. The parallel loss of strength in both bone and skeletal muscle tissues has been termed osteosarcopenia [6]. Fundamental to the concept of osteosarcopenia is that this parallel degeneration in the bone and skeletal muscle tissues is not coincidence, rather that these tissues are communicating with each other in a negative feedback loop that contribute to overall musculoskeletal weakness that contributes to poor clinical outcomes [7]. However, our understanding of this inter-tissue crosstalk between bone and muscle in osteosarcopenia remains limited.

Bone–muscle crosstalk is a growing field of interest, as studies have identified the importance of biochemical crosstalk between these two tissues in a variety of contexts [8]. Yet, molecular mechanism(s) associated with this interaction are not fully understood. One known method of bone muscle crosstalk is the exchange of microRNAs (miRs) between bone and skeletal muscle cells [9]. miRs are known to be transported via exosomes to other tissues, and in particular the osteocyte is known to release exosomes [10]. Previous work has defined a critical role for the osteocyte, cells embedded within the bone matrix, in maintaining bone and facilitating bone–muscle crosstalk [11]. Changes in the osteocyte have also been shown to contribute to bone loss and bone fragility. In particular, osteocyte apoptosis has been shown to play a critical role in age-related bone loss via loss of connexin43 (Cx43) expression [12–14]. These changes to the osteocyte have deleterious effects on both the bone geometry and strength, and previous studies have suggested this may be mediated through decreases in microRNA 21 (miR21) [15]. miR21 is known to be a pro-survival microRNA, and expression of miR21 decreases with advancing age in osteocytes from mice, suggesting it contributes to osteocyte apoptosis with aging [16]. However, selective deletion of miR21 from the osteocyte did not mimic age-related bone loss and weakness. Rather, male and female mice lacking osteocytic miR21 (OtmiR21Δ) had increased bone strength, in conjunction with sexually dimorphic changes in the osteocytic secretome that had differing effects on osteoclasts and osteoblasts [17]. Changes in the osteocyte secretome have previously been shown to contribute to bone–muscle crosstalk and led us to investigate further whether osteocytic miR21 deletion had an effect on skeletal muscle.

Our previous studies showed that deletion of osteocytic miR21 leads to changes in the pattern of gene expression/protein secretion by bone and altered osteoclast differentiation in a sex-dependent manner [17]. Herein, we propose to test the hypothesis that, similarly, osteocytic miR21 regulates skeletal muscle mass and function. We found that female animals lacking osteocytic miR21 have increased percent lean mass and relative skeletal muscle weight, but no change in fiber cross-sectional area or strength production. Changes in skeletal muscle mass/function are associated with higher T185/Y187-ERK1/2 phosphorylation in miR21-deficient females and lower and higher S473-Akt phosphorylation, respectively, in female and male OtmiR21Δ mice compared to miR21fl/fl controls. Male animals lacking osteocytic miR21 do not have changes in percent lean mass or skeletal muscle weight, but significantly higher time to ½ relaxation and expression of genes associated with protein catabolism. C2C12 myotubes increased in diameter when exposed to conditioned media from bones of female mice lacking osteocytic miR21 compared to miR21fl/fl controls, while no effect was seen in male mice, suggesting bone–muscle crosstalk may explain these sex-dependent effects of osteocytic miR21 deletion on skeletal muscle.

## Methods

### Mice

miR21 floxed mouse strain was engineered with lox cassettes on both sides of the mmu-miR-21 genomic locus (named miR21fl/fl) [15], and crossed with DMP1-8 kb-Cre mice [18] to obtain female and male miR21fl/fl and miR21fl/fl;DMP1-8 kb-Cre (OtmiR21Δ) mice [19]. Four-month-old female and male littermate mice were analyzed. All mice were of the C57BL/6 background, fed a regular diet and water ad libitum and maintained on a 12-h light/dark cycle. Mice were genotyped by PCR using genomic DNA extraction from mouse ear notches and genotyped using primers as previously described [15]. At 4 months of age, food was removed, and mice were euthanized by isoflurane overdose and cervical dislocation 3 h later. Calvaria bone and himdlimb skeletal muscles were collected, snapped frozen, and stored at -80 °C until used, or prepared for organ culture as detailed below. The protocols involving mice were approved by the Institutional Animal Care and Use Committee of Indiana University School of Medicine.

### qPCR

Total RNA was isolated using TRIzol (Invitrogen, Grand Island, NY) [20]. Expression levels of miR21 (assay ID:000397) and the house-keeping miR135 (assay ID:001230) were evaluated using Applied Biosystem reagents, as published [15]. No differences were detected between genotypes in miR135 Ct values for either female or male mice (Ct values: female miR21fl/fl, 35.0 ± 0.5; female ΔOtmiR21, 34.3 ± 1.0; 19.6 ± 1.4; male miR21fl/fl, 33.7 ± 0.6; male ΔOtmiR21, 35.0 ± 0.7). Reverse transcription was performed using a high-capacity cDNA kit (Applied Biosystems, Foster City, CA). qPCR was performed using the Gene Expression Assay Mix TaqMan Universal Master Mix with the 7500 Real Time PCR/StepOne Plus system and software (Life Technologies). Gene expression was corrected by the levels of the house-keeping gene glyceraldehyde 3-phosphate dehydrogenase (GAPDH), which showed Ct values that did not differ among groups as assessed by 2-way ANOVA (Ct values: female miR21fl/fl, 20.3 ± 1.6; female ΔOtmiR21, 19.6 ± 1.4; male miR21fl/fl, 19.3 ± 1.1; male ΔOtmiR21, 18.3 ± 0.8). Primers and probes were commercially available (Applied Biosystems, Foster City, CA) or were designed using the Assay Design Center (Roche Applied Science, Indianapolis, IN, USA) (Additional file 1: Table S1). Relative expression was calculated using the ∆Ct method.

### Body weight and body composition by dual-energy X-ray absorptiometry (DXA)

DXA/PIXImus scans were performed in 4-month-old mice (G.E. Medical Systems, Lunar Division, Madison, WI, USA) [20] a day prior to euthanizing the mice. Body weight was measured at the time of the DXA scan. Calibration was performed using a standard control phantom before scanning, as recommended by the manufacturer. The total tissue mass (TTM) measurement was used to calculate the fat percentage (total fat body mass (g)/tissue total mass) and lean percentage (total lean body mass (g)/tissue total mass) as previously described [21].

### Grip strength

The evaluation of the whole body strength in mice was assessed as previously described [22] one week before euthanizing the mice. The absolute grip strength (peak force, expressed in grams) was recorded by means of a grip strength meter (Columbus Instruments, Columbus, OH, USA) and corrected by the corresponding body weight (BW) to render normalized force. Five measurements were completed, and the top three measurements were included in the analysis.

### In vivo muscle contractility

A separate cohort of mice was tested for muscle force by in vivo plantarflexion (Aurora Scientific, Aurora, ON, Canada), as described previously [23, 24]. Briefly, the left hind foot was taped to the force transducer and positioned to where the foot and tibia were aligned at 90°. The knee was then clamped at the femoral condyles, avoiding compression of the fibular nerve. Two disposable monopolar electrodes (Natus Neurology, Middleton, WI, USA) were placed subcutaneously posterior/medial to the knee in order to stimulate the tibial nerve. Peak twitch torque was first established in order to determine maximal stimulus intensity. Plantarflexion force was measured following stimulation at 100 Hz, and corrected by the weight of the corresponding mouse.

### Multiplex cell-signaling assays

Cell-signaling pathway alterations induced by deletion of osteocytic miR21 were examined in miR21fl/fl and OtmiR21Δ soleus skeletal muscle lysates, prepared following the instructions from the Milliplex multi-pathway 9-plex phospho- and total protein kits (Millipore Sigma catalog # 48-680MAG and 48-681MAG, respectively), as previously reported [17]. Phospho-cAMP response element-binding protein, CREB (pS133), extracellular-regulated signal kinase, ERK1/2 (pT185/pY187), nuclear factor kappa-light-chain-enhancer of activated B cells, NFκB (pS536), c-Jun N-terminal kinase, JNK (pT183/pY185), p38 mitogen-activated protein kinase, p38 (Thr180/Tyr182), ribosomal protein S6 kinase beta-1, p70S6K (Thr412), signal transducer and activator of transcription STAT3 (pS727) and STAT5A/B (pY694/699), and protein kinase B (Akt pS473) as well as total protein levels for each kinase were measured.

### Ex vivo bone organ cultures

Long bones were isolated from male and female 4-month-old miR21fl/fl and OtmiR21Δ mice. Bone-marrow cells (BMCs) were flushed out with α-MEM and osteocyte-enriched long bones were cultured ex vivo in 10% FBS and 1% penicillin/streptomycin (P/S)-α-MEM supplemented for 48 h. Conditioned media were collected and stored at -20 °C until used for the C2C12 assays.

### Assessment of muscle cross-sectional area (CSA)

Ten-μm-thick cryosections of gastrocnemius muscles taken at the mid-belly were processed for immunostaining [22]. Samples were marked with a histology marking pen, blocked in phosphate buffered saline (PBS) containing 8% bovine serum albumin (BSA) for 1 h at room temperature, and incubated at 4 °C overnight with dystrophin primary antibody (1:200 in 8% BSA, Developmental Studies Hybridoma Bank, Iowa City, IA; #MANDRA1(7A10)). After the overnight incubation, samples were washed prior to incubation with a secondary antibody (1:500 in 8% BSA, ThermoFisher Scientific; AlexaFluor 555, #A-11032) for 1 h. Samples were then washed with PBS and mounted with ProLong Antifade mounting medium (ThermoFisher Scientific). For determination of the CSA, the entire muscle section was imaged and quantified by using the Lionheart XL microscope system and the Gen5 software (BioTek, Winooski, VT, USA).

### C2C12 myotube differentiation

Murine C2C12 skeletal myoblasts (ATCC, Manassas, VA, USA) were grown in high glucose DMEM supplemented with 10% FBS, 100 U/ml penicillin, 100 mg/ml streptomycin, 2 mM l-glutamine, and maintained at 37 °C in 5% CO_2_, as previously published [25]. Myotubes were generated by exposing the myoblasts to DMEM containing 2% horse serum (i.e., differentiation medium), and replacing the medium every other day for 5 days. In order to determine the dependence of myotube size on bone-derived factors, myotubes were exposed to 5% bone conditioned medium (CM) for 48 h. Cells were fixed and stained [26, 27].

### Assessment of myotube size

C2C12 cell layers were fixed in ice-cold acetone–methanol and incubated with an anti-Myosin Heavy Chain antibody (MF-20, 1:200; Developmental Studies Hybridoma Bank, Iowa City, IA, USA) and an AlexaFluor 488-labeled secondary antibody (Invitrogen, Grand Island, NY, USA), as reported in [26, 27]. Analysis of myotube size was performed by measuring the minimum diameter of long, multi-nucleated fibers avoiding regions of clustered nuclei on a calibrated image using the Image J 1.43 software [28]. Images were taken using a Axio Observer.Z1 motorized fluorescence microscope (Zeiss, Oberchoken, Germany). Three biological replicates (*n* = 3) were generated for each experimental condition, and about 200–350 myotubes per replicate were measured. The results of each replicate were then averaged to obtain the final myotube size.

### Statistics

All statistical analyses were performed using SigmaPlot (Systat Software Inc., San Jose, CA). Data are reported as mean ± SD and as individual values. Data were evaluated by two-way ANOVA, followed by All Pairwise Multiple Comparison Procedures (Holm–Sidak method). Statistical significance was set at *p* < 0.05.

## Results

**Fig. 1:**
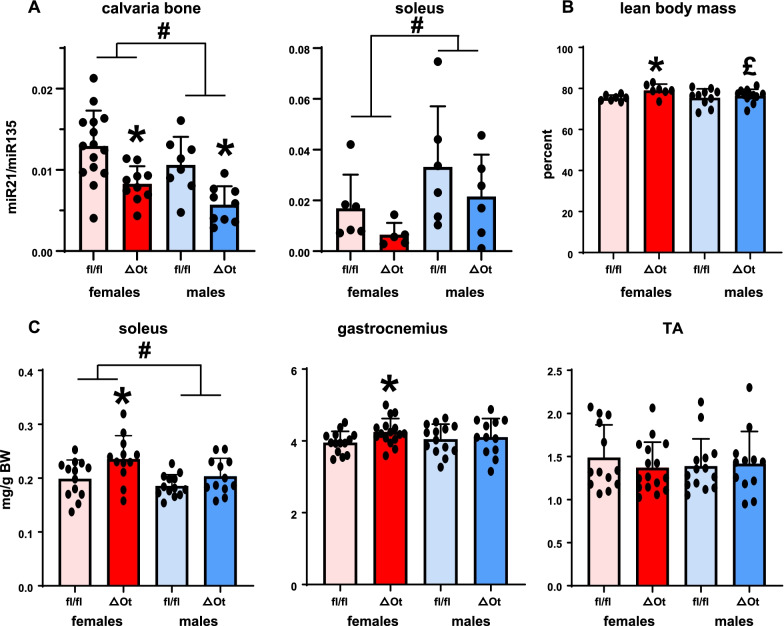
Female OtmiR21Δ mice have increased lean mass by DXA/Piximus and muscle weights compared to miR21fl/fl controls. miR21 expression corrected by miR135 levels in calvaria bone (N = 8–15/group) and soleus muscles (N = 5–6/group) (**A**), percent lean body mass (N = 5–7/group) (**B**), and muscle wet weight corrected by body weight (**C**) of female and male OtmiR21Δ and miR21fl/fl mice (N = 12–16/group). Two-way ANOVA analyses were used to determine significant differences (Additional file 2: Table S2) *p < 0.05 compared to sex-matched miR21fl/fl controls, #*p* < 0.05 for overall male versus female comparisons, and £*p* < 0.05 compared to females of the same genotype

As expected for an osteocyte-rich tissue, miR21 levels were significantly lower in both female (− 36%) and male (− 46%) mice lacking miR21 in the osteocyte (OtmiR21Δ) compared to littermate controls (miR21fl/fl) in the calvaria (Fig. 1A). On the other hand, the levels of miR21 in soleus muscle lysates from male and female OtmiR21Δ mice were not significantly different. miR21 levels were higher (~ 1.3-fold) in calvaria bone but lower in soleus muscle (~ 0.4-fold) in females compared to males, resulting in an overall sex effect for both organs (Additional file 2: Table S2). Deletion of osteocytic miR21 did not affect miR21 in the tibialis anterior with neither sex nor genotype altering miR21 levels (Additional file 3: Fig. S1; Additional file 2: Table 2). On the other hand, miR21 was lower in the gastrocnemius of both female (− 53%) and male (− 82%) OtmiR21Δ muscles compared to miR21fl/fl of the same sex. Statistical analysis showed that whereas there was an overall sex effect on gastrocnemius miR21 levels, the differences between female and male mice only reached significance for control miR21fl/fl mice.

Female, but not male, OtmiR21Δ mice have higher percent lean mass compared to miR21fl/fl controls (+ 5%) as measured by DXA/Piximus (Fig. 1B; Additional file 2: Table S2). Female OtmiR21Δ mice also have higher wet weight of the soleus (18%) and gastrocnemius (7%) muscles corrected by total body weight, compared to miR21fl/fl controls, whereas only soleus muscle weight showed an overall sex effect, with female weight/body weight ~ 11% higher than males (Fig. 1C; Additional file 2: Table S2). No differences in normalized skeletal muscle weights were found in male mice. On the other hand, the tibialis anterior (TA) muscle mass (corrected by body weight) was similar in all animals, independent of the genotype or sex.

**Fig. 2:**
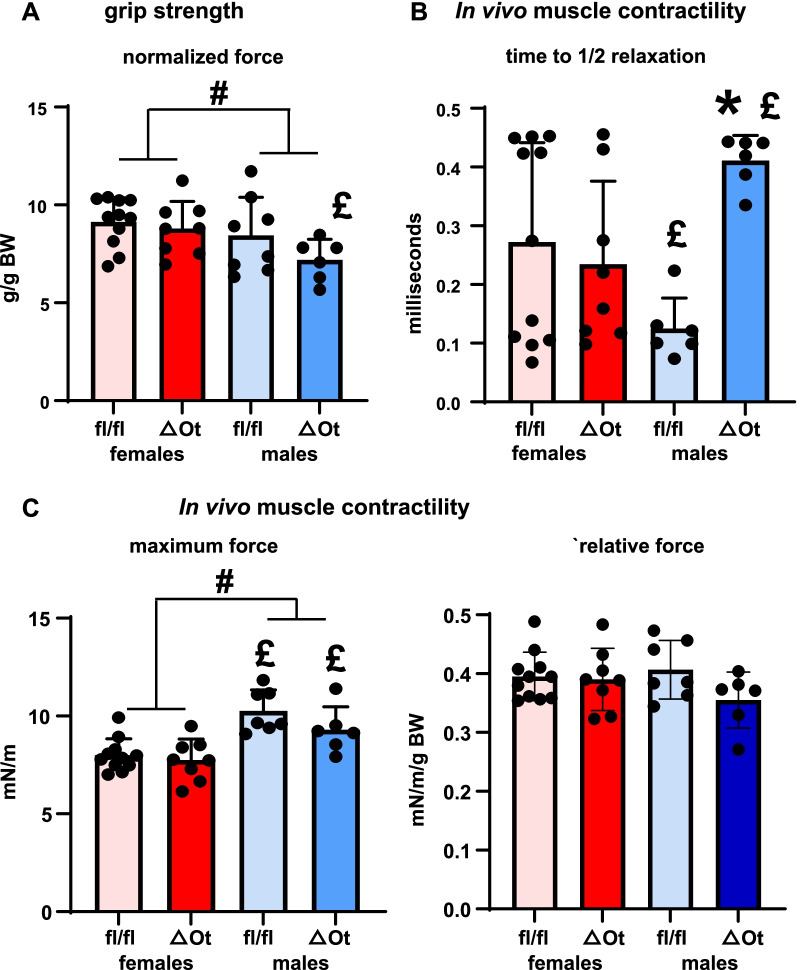
Sex, but not genotype, affects grip strength and maximum force, whereas deletion of osteocytic miR21 only affects time to ½ relaxation in male OtmiR21Δ mice. In vivo grip strength (**A**) and plantar flexion (**B**, **C**) measurements from female and male OtmiR21Δ and miR21fl/fl mice (N = 6–11/group). Two-way ANOVA analyses were used to determine significant differences (Additional file 2: Table S2) *p < 0.05 compared to sex-matched miR21fl/fl controls, #*p* < 0.05 for overall male versus female comparisons, and £*p* < 0.05 compared to females of the same genotype

Although female OtmiR21Δ mice showed higher skeletal muscle mass, neither female nor male mice demonstrated changes in whole body grip strength between genotypes (Fig. 2A). Yet, grip strength was overall higher in females, and female OtmiR21Δ exhibited 22% higher grip strength than males of the same genotype, with no changes for miR21fl/fl mice (Additional file 2: Table S2). On the other hand, while male OtmiR21Δ mice did demonstrate a 3.3-fold higher time to ½ relaxation compared to miR21 fl/fl mice, male mice of both genotypes showed significantly different time to ½ relaxation compared to the respective female mice, which was 0.45-fold lower in miR21fl/fl and 1.75-fold higher in OtmiR21Δ mice (Fig. 2B, Additional file 2: Table S2). In contrast, plantar flexion in female or male OtmiR21Δ mice did not show changes in maximum force compared to miR21fl/fl (Fig. 2C; Additional file 2: Table S2), but there was a significant sex effect with males showing higher strength (~ 24%) compared to females of the respective genotypes. The sex differences were lost when the force was corrected by body weight.

**Fig. 3:**
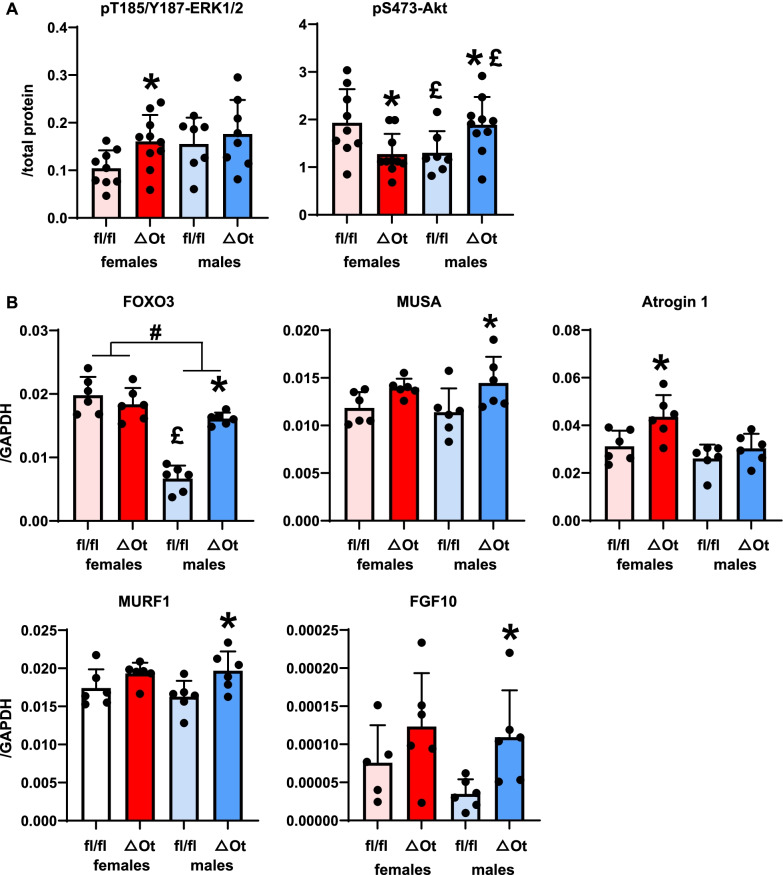
Sex-dependent alterations in kinase phosphorylation and gene expression in soleus muscles from OtmiR21Δ and miR21fl/fl mice. Multiplex protein analysis of phosphorylated and total kinase levels (N = 7–10/group) (**A**) and mRNA expression corrected by GAPDH levels (N = 5–6) (**B**) of soleus muscle lysates from female and male OtmiR21Δ and miR21fl/fl mice. Two-way ANOVA analyses were used to determine significant differences (Additional file 2: Table S2) *p < 0.05 compared to sex-matched miR21fl/fl controls, #*p* < 0.05 for overall male versus female comparisons, and £*p* < 0.05 compared to females of the same genotype

To further explore the potential mechanism underlying these sex-dimorphic effects of miR21 deletion in osteocytes, the levels of protein and mRNA were measured in the soleus muscles from male and female OtmiR21Δ and miR21fl/fl animals. Simultaneous analysis of phospho- and total protein levels showed that whereas protein was detected for all kinases, phosphorylation was only detectable for JNK, ERK1/2, Akt, and p38 (not shown). Of those, miR21 deletion did not affect phospho/total JNK and p38 levels in females or males (data not shown). On the other hand, the ratio of phosphorylated ERK1/2 (pT185/pY187) and Akt (S473) to total protein levels for these kinases known to be involved in skeletal muscle mass and homeostasis [29, 30], was, respectively, 54% higher and 33% lower in female OtmiR21Δ mice compared to miR21fl/fl controls (Fig. 3A). Interestingly, male OtmiR21Δ mice showed 45% higher Akt phosphorylation compared to miR21fl/fl controls, but no differences were seen in T185/Y187-ERK1/2 phosphorylation. Statistical analysis also showed a significant genotype effect and a tendency towards sex effects in ERK1/2 phosphorylation (*p* = 0.068) without sex–genotype interactions. On the other hand, there was a significant sex–genotype interaction in phospho-S473/total Akt levels, with values in soleus muscle 33% lower and 48% higher in control and osteocytic miR21-deficient mice, respectively, compared to the corresponding female mice. Because these kinases are known to be associated with protein catabolism pathways, we decided to investigate whether genes involved in protein catabolism were also impacted by the deletion of miR21 in osteocytes.

Akt and its downstream target the transcription factor FOXO3 control the expression of E3 ubiquitin ligases, which are, in turn, known to regulate protein catabolism. We found that female OtmiR21Δ mice do not exhibit changes in FOXO3 and only demonstrated higher mRNA levels of the E3 ubiquitin ligase Atrogin 1 compared to miR21fl/fl controls in soleus muscle (Fig. 3B). On the other hand, male OtmiR21Δ mice had higher mRNA levels of FOXO3 and its downstream E3 ubiquitin ligases MUSA and MuRF-1. Regarding the sex effects on gene expression, we found an overall female–male difference in FOXO3 mRNA levels, which were 77% lower only in miR21fl/fl males compared to females of the same genotype. Taken together, these data suggest that ubiquitin ligases are upregulated differentially in both male and female OtmiR21Δ mice in response to miR21 deletion, potentially as a consequence of differential Akt/ERK1/2 activation.

ERK1/2 activity was shown to be regulated by the fibroblast growth factor 10 (FGF10), though not much is known about the role of this factor in skeletal muscle homeostasis. [31]. mRNA levels of FGF10 were 3.1-fold higher only in male OtmiR21Δ mice compared to miR21fl/fl controls, whereas ERK1/2 activity was higher in female but not male OtmiR21Δ mice (Fig. 3B), suggesting other mechanisms are involved in the regulation of FGF10 in the absence of osteocytic miR21.

**Fig. 4:**
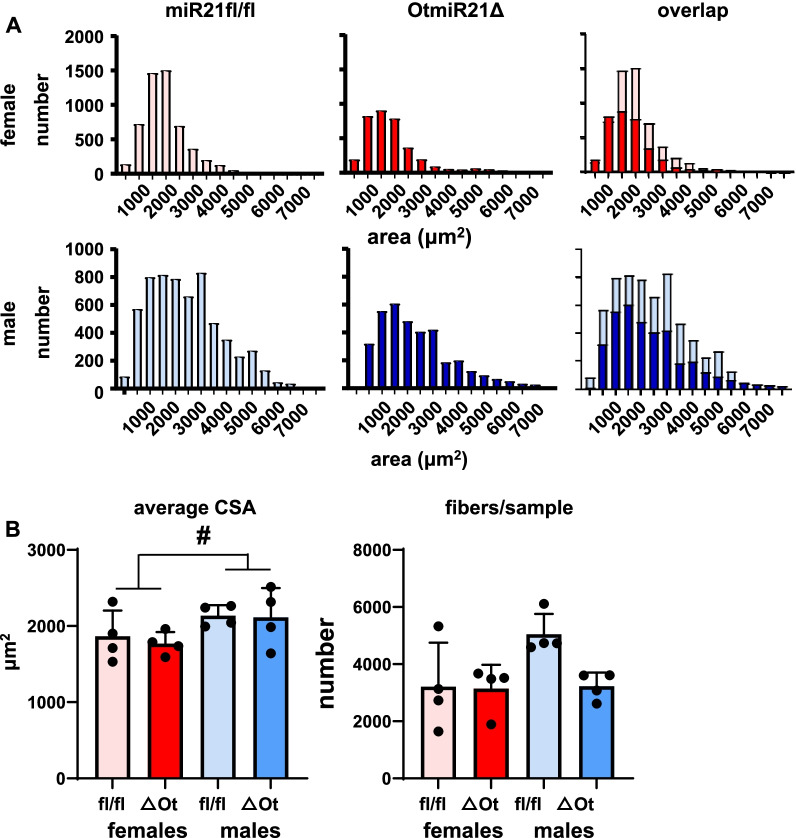
Sex, but not deletion of osteocytic miR21, causes differences in gastrocnemius muscle cross-sectional area (CSA) in OtmiR21Δ and miR21fl/fl mice. Gastrocnemius skeletal muscle CSA from female and male OtmiR21Δ and miR21fl/fl mice (N = 4/group). **A** Frequency distribution for fibers measured in muscles from female and male mice. Graphs on the right show the overlap of the fiber area for both genotypes. **B** Average CSA and number of fibers measured/sample. Two-way ANOVA analyses were used to determine significant differences (Additional file 2: Table S2). #*p* < 0.05 for overall male versus female comparisons

To assess whether the changes in muscle mass were associated with alterations in skeletal muscle fiber area, we measured the cross-sectional area (CSA) of fibers from the gastrocnemius muscles. No differences in the distribution of CSAs were found for either sex as demonstrated in overlaid histograms (Fig. 4A), in spite of the lower levels of miR21 in these muscles (Additional file 3: Fig. S1). Further, osteocytic miR21 deletion did not alter the average CSA or the number of fibers in each sample (Fig. 4B). On the other hand, the average CSA was 14 and 20% higher for miR21fl/fl and OtmiR21Δ mice, respectively, in muscles from males than females. These data suggest that there must be some other contributing factor to the observed higher skeletal muscle wet weight of female OtmiR21Δ mice compared to miR21fl/fl that remains unknown.

**Fig. 5:**
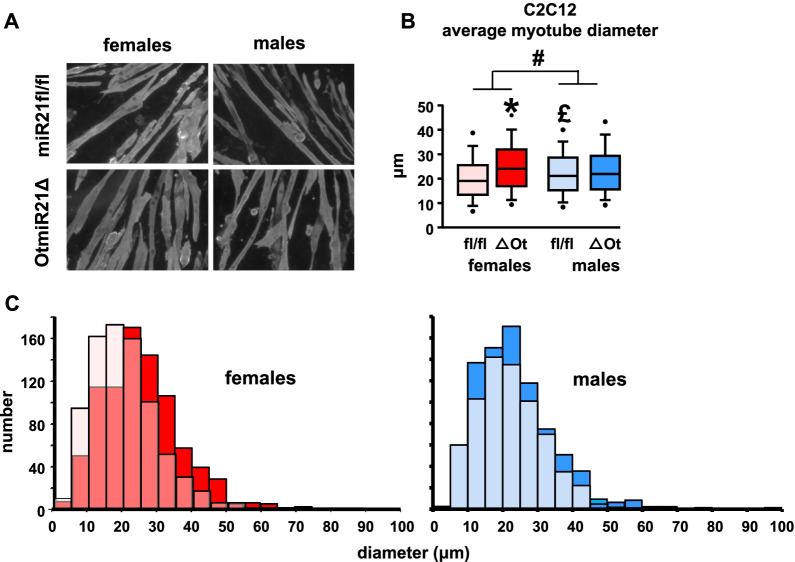
Only conditioned media (CM) from female OtmiR21Δ mice increases myotube diameter. Representative images (**A**) and quantification of myotube diameter (**B**) in C2C12 cultures exposed to CM made from bones of female and male OtmiR21Δ and miR21fl/fl mice (N = 3 biological replicas/group). **C** Frequency distributions of the diameter of 200 to 350 myotubes measured. Each graph shows the diameter of C2C12 cells treated with CM for females or males of both genotypes. Two-way ANOVA analyses were used to determine significant differences (Additional file 2: Table S2) *p ≤ 0.05 compared to sex-matched miR21fl/fl controls, #*p* < 0.05 for overall male versus female comparisons, and £*p* < 0.05 compared to females of the same genotype

Therefore, to assess whether bone–muscle crosstalk contributes to these changes in skeletal muscle, conditioned media were made from marrow-flushed bones of female and male OtmiR21Δ and miR21fl/fl mice. Addition of conditioned media from female OtmiR21Δ bones for 48 h led to higher C2C12 myotube diameter by 12% compared to cells treated with conditioned media from miR21fl/fl controls (Fig. 5A, B). The effect of the conditioned media is also demonstrated by the rightward shift in the frequency distribution of myotube sizes (Fig. 5C). On the other hand, no difference was seen in C2C12 myotubes exposed to conditioned media from male OtmiR21Δ compared to miR21fl/fl bones. In addition, although there was an overall sex effect in the myotube diameter, the post hoc test showed significant differences only in miR21fl/fl male mice compared to females of the same genotype (Fig. 5B). These data suggest that the sex-specific skeletal muscle alternations in the OtmiR21Δ mice do not depend on the direct actions of the microRNA in the muscle.

## Discussion

Herein, we report higher percentage of lean mass in female OtmiR21Δ mice compared to miR21fl/fl controls, but not in males. These changes in lean body mass are complemented by larger gastrocnemius and soleus muscles, but not tibialis anterior, of female OtmiR21Δ mice compared to miR21fl/fl. However, assessment of grip strength and plantar flexion force demonstrated that the deletion of miR21 had no effect on strength. The concurrent observations of augmented skeletal muscle mass without changes in strength are not new. Although skeletal muscle strength and mass are typically correlated in many cases, it has been previously shown that genetic knockdown of myostatin increases skeletal muscle mass with no effect on total force production [32, 33]. Further, hypertrophy in the absence of increased strength is typically due to other cellular or molecular changes such as changes in calcium handling in the case of hypertrophy caused by mutations in myostatin [34]. However, other aspects of skeletal muscle function beyond force production may be altered, as our data show time to relaxation is significantly increased in male OtmiR21Δ animals compared to miR21fl/fl even though lean mass and muscle wet weight remains unaltered.

A primary limitation of this study is the DMP1-8 kb expression in skeletal muscle leading to a tendency towards lower miR21 expression in skeletal muscle lysates from both male and female OtmiR21Δ mice. These data may be explained by previous reports of DMP1-8 kb activity in skeletal muscle [35, 36]. However, miR21 expression in the soleus muscle lysates was significantly lower than expression in bone as demonstrated by an average CT value in female and male miR21fl/fl bones (31.4 and 33.5) compared to soleus (36.7 and 37.0). Additionally, C2C12 myotubes exposed to conditioned media from female OtmiR21Δ marrow-flushed bones did exhibit higher myotube diameter, in line with the higher skeletal muscle mass seen in females as well. These suggest that, even if miR21 is expressed in soleus skeletal muscle, loss of miR21 expression specifically from bone has a measurable impact on skeletal muscle in ex vivo cell culture. Therefore, it seems that although miR21 expression may be altered in the skeletal muscle of OtmiR21Δ mice, the phenotype is still a result of bone–muscle crosstalk.

Multiplex assessment of protein lysates from the soleus muscle demonstrated that female OtmiR21Δ mice had higher ERK1/2 (T185/Y187) and lower Akt (S473) phosphorylation compared to miR21fl/fl controls. These data seem counterintuitive to what is known about Akt signaling in skeletal muscle, which is known to promote skeletal muscle anabolism through activation of mTOR [37]. However, beyond mTOR, Akt activity is known to inhibit FOXO-mediated transcription of atrophy-related ubiquitin ligases such as Atrogin-1 and MuRF-1 [38]. Although mRNA levels of FOXO3 were not changed in female OtmiR21Δ compared to miR21fl/fl, mRNA expression of Atrogin-1 was higher compared to miR21fl/fl littermates, in line with lower S473-Akt phosphorylation. Of note, miR21 deletion is also known to downregulate Akt signaling via increased PTEN, potentially explaining these data [39]. Further studies are needed to fully understand the signaling activated downstream of Akt in the context of reduced osteocytic miR21 levels, and whether sex influences the expression or activity of those signaling molecules.

Further, previous work in this animal model of osteocytic miR21 deletion showed a decrease in osteoclastic resorption in female but not male miR21-deficient animals, suggesting that decreases in bone matrix-released products may have a positive effect on skeletal muscle mass as demonstrated in vivo in Fig. 1B, C. Bone-matrix-derived products such as TGF-β have previously been shown to be released from the bone during resorption and have a deleterious effect on skeletal muscle [40]. Further, decreasing bone resorption by bisphosphonates rescues the skeletal muscle mass in models of cancer and chemotherapy cachexia [40, 41]. These previous studies may explain why conditioned media derived from bones of female OtmiR21Δ mice led to increased myotube size compared to miR21fl/fl, but no effect on muscle size was seen with conditioned media from males. However, male OtmiR21Δ animals exhibit higher S473-Akt phosphorylation and FOXO3 and MuRF-1 mRNA levels in the soleus muscle. These data suggest that either downstream Akt signaling is being inhibited by deletion of miR21 or that FOXO3 upregulates MuRF-1 mRNA levels in the absence of miR21 independent of Akt.

mRNA levels of FGF10 were also higher in male but not in female OtmiR21Δ mice, suggesting that FGF10 may be upregulating FOXO3 expression as previously shown in cardiomyocytes [42]. This would suggest that although Akt activity is elevated in male OtmiR21Δ mice, FGF10 may be upregulating FOXO3, which is known to increase transcription of MuRF-1. FGF10 function in skeletal muscle beyond development is not well understood, and further work is needed to understand the role of FGF10 in skeletal muscle, and how osteocytic miR21 may contribute to these changes.

### Perspectives and significance

Overall, our study provides insight into the effect of bone-targeted deletion of osteocytic microRNAs on skeletal muscle and, in particular, into the molecular mechanisms that govern skeletal muscle mass and strength in the absence of osteocytic miR21. Here we found that, instead of reducing muscle mass or strength, deletion of miR21 in osteocytes increases them, thus allowing us to speculate that factors other than miR21 mediate the role of bone on skeletal muscle aging. Alternatively, it is possible that consequences of aging intrinsic to skeletal muscle, rather than changes in factors released by bone cells and osteocytes in particular, contribute to sarcopenia.

Our study also offers a side-by-side comparison of the role of sex on the consequences of the genetic manipulation, emphasizing the need to investigate both females and males when describing the mouse phenotypes. Future studies will be needed to specifically address the skeletal muscle-intrinsic effect of miR21 on skeletal muscle fiber types in a sex-specific context and, specifically, to determine if, as with females, bone miR21 levels change in males with old age, thus also contributing to the onset of sarcopenia.

## Supplementary Information

**Additional file 1**: **Table S1. **qPCR primer/probe set Applied Biosystems (ABI) Assay-on-Demand ID number or primer sequence for the genes analyzed in Fig. 3B.

**Additional file 2**: **Table S2. **Two-way ANOVA analyses of the data included in the manuscript. Post hoc analyses are only indicated for comparisons with p<0.05 for the corresponding source of variation. Pairwise comparisons with p<0.05 are in bold text.

**Additional file 3**: **Fig. S1** Expression of miR21 in gastrocnemius and tibialis anterior (TA) muscles. Expression of miR21 corrected by miR135 levels in the gastrocnemius and TA muscles (N = 5–6/group) from female and male OtmiR21Δ and miR21fl/fl mice. Two-way ANOVA analyses were used to determine significant differences (Additional file 2: Table S2) * = p ≤ 0.05 compared to sex-matched miR21fl/fl controls, # = p < 0.05 for overall male versus female comparisons, and £ = p < 0.05 compared to females of the same genotype.

## Data Availability

The datasets during and/or analyzed during the current study are available from the corresponding author on reasonable request.
