# Supplementary material for: miR21 deletion in osteocytes has direct and indirect effects on skeletal muscle in a sex-dimorphic manner in mice
Source: Biol Sex Differ. 2022 Oct 1;13:56. doi: 10.1186/s13293-022-00465-9 (PMC9526971; doi:10.1186/s13293-022-00465-9)
Supplement: Supplementary file 1 — Additional file 1: Table S1. qPCR primer/probe set Applied Biosystems (ABI) Assay-on-Demand ID number or primer sequence for the genes analyzed in Fig. 3B. [file 13293_2022_465_MOESM1_ESM.docx]

**Supplementary Table 1.** qPCR primer/probe set Applied Biosystems (ABI) Assay-on-Demand ID number or primer sequence for the genes analyzed in **Figure 3B**.

| **gene** | **vendor** | **primer ID** |  |  |
| --- | --- | --- | --- | --- |
| GAPDH | ABI | Mm03302249_g1 |  |  |
|  |  |  |  |  |
| **gene** | **vendor** | **Primer Sequence (forward)** | **Primer Sequence (reverse)** | **Universal Probe (Millipore Sigma)** |
| Foxo3 | Roche | gctaagcaggcctcatctca | ttccgtcagtttgagggtct | #64 (cat. no. 04688635001) |
| MUSA1 | Roche | gagaagccagggtttgagc | tcatacagtgtgagtgctgctg | #69 (cat. no. 04688686001) |
| Atrogin1 | Roche | agtgaggaccggctactgtg | gatcaaacgcttgcgaatct | #53 (cat. no. 04688503001) |
| Murf1 | Roche | cctgcagagtgaccaagga | ggcgtagagggtgtcaaact | #17 (cat. no. 04686900001) |
| FGF10 | Roche | cgggaccaagaatgaagact | gcaacaactccgatttccac | #80 (cat. no.04689038001) |
