# Supplementary material for: miR21 deletion in osteocytes has direct and indirect effects on skeletal muscle in a sex-dimorphic manner in mice
Source: Biol Sex Differ. 2022 Oct 1;13:56. doi: 10.1186/s13293-022-00465-9 (PMC9526971; doi:10.1186/s13293-022-00465-9)
Supplement: Supplementary file 2 — Additional file 2: Table S2. Two-way ANOVA analyses of the data included in the manuscript. Post hoc analyses are only indicated for comparisons with p<0.05 for the corresponding source of variation. Pairwise comparisons with p<0.05 are in bold text. [file 13293_2022_465_MOESM2_ESM.docx]

**Supplementary Table 2.** Two-way ANOVA analyses of the data included in the manuscript. Post-hoc analyses are only indicated for comparisons with p<0.05 for the corresponding source of variation. Pairwise comparisons with p<0.05 are in bold text.

| **Figure/panel** | **endpoint** | **source of variation** | **All Pairwise Multiple Comparison Procedures (Holm-Sidak method)** |
| --- | --- | --- | --- |
| **1A** | miR21- calvaria bone | Genotype <0.001  Sex 0.028  Genotype x Sex 0.914 | **genotype within female: p<0.05**  **genotype within male: p<0.05**  sex within fl/fl: p>0.05  sex within ΔOt: p>0.05 |
|  | miR21- soleus | Genotype 0.127  Sex 0.035  Genotype x Sex 0.926 | sex within fl/fl: p>0.05  sex within ΔOt: p>0.05 |
| **1B** | lean body mass | Genotype 0.006  Sex 0.546  Genotype x Sex 0.003 | **genotype within female: p<0.05**  genotype within male: p>0.05  sex within fl/fl: p>0.05  **sex within ΔOt: p<0.05** |
| **1C** | soleus corrected weight | Genotype 0.006  Sex 0.022  Genotype x Sex 0.330 | **genotype within female: p<0.05**  genotype within male: p>p0.05 |
|  | gastrocnemius corrected weight | Genotype 0.031  Sex 0.668  Genotype x Sex 0.093 | **genotype within female: p<0.05**  genotype within male: p>0.05 |
|  | TA corrected weight | Genotype 0.626  Sex 0.782  Genotype x Sex 0.729 | N/A |
| **2A** | grip strength | Genotype 0.132  Sex 0.034  Genotype x Sex 0.378 | sex within fl/fl: p>0.05  **sex within ΔOt: p<0.05** |
| **2B** | time to ½ relaxation | Genotype 0.015  Sex 0.763  Genotype x Sex 0.002 | genotype within female: p>0.05  **genotype within male: p<0.05**  **sex within fl/fl: p<0.05**  **sex within ΔOt: p<0.05** |
| **2C** | maximum force | Genotype 0.109  Sex 0.001  Genotype x Sex 0.336 | **sex within fl/fl: p<0.05**  **sex within ΔOt: p<0.05** |
|  | relative force | Genotype 0.110  Sex 0.498  Genotype x Sex 0.185 | N/A |
| **3A** | ERK_1/2_ | Genotype 0.039  Sex 0.068  Genotype x Sex 0.286 | **genotype within female: p<0.05**  genotype within male: p>0.05 |
|  | Akt | Genotype 0.842  Sex 0.975  Genotype x Sex 0.002 | **genotype within female: p<0.05**  **genotype within male: p<0.05**  **sex within fl/fl: p<0.05**  **sex within ΔOt: p<0.05** |
| **3B** | Foxo3 | Genotype <0.001  Sex <0.001  Genotype x Sex <0.001 | genotype within female: p>0.05  **genotype within male: p<0.05**  **sex within fl/fl: p<0.05**  sex within ΔOt: p>0.05 |
|  | MUSA | Genotype 0.007  Sex 0.985  Genotype x Sex 0.587 | genotype within female: p>0.05  **genotype within male: p<0.05** |
|  | Atrogin 1 | Genotype 0.009  Sex 0.004  Genotype x Sex 0.173 | **genotype within female: p<0.05**  genotype within male: p>0.05  sex within fl/fl: p>0.05  **sex within ΔOt: p<0.05** |
|  | MURF1 | Genotype 0.007  Sex 0.669  Genotype x Sex 0.411 | genotype within female: p>0.05  **genotype within male: p<0.05** |
|  | FGF10 | Genotype 0.014  Sex 0.238  Genotype x Sex 0.555 | genotype within female: p>0.05  **genotype within male: p<0.05** |
| **4B** | CSA | Genotype 0.675  Sex 0.045  Genotype x Sex 0.789 | sex within fl/fl: p>0.05  sex within ΔOt: p>0.05 |
|  | fibers per sample | Genotype 0.078  Sex 0.073  Genotype x Sex 0.099 | N/A |
| **5B** | myotube diameter | Genotype 0.005  Sex 0.018  Genotype x Sex 0.576 | **genotype within female: p<0.05**  genotype within male: p>0.05  **sex within fl/fl: p<0.05**  sex within ΔOt: p>0.05 |
| **Suppl. 1** | miR21 - gastrocnemius | Genotype <0.001  Sex 0.004  Genotype x Sex 0.327 | **genotype within female: p<0.05**  **genotype within male: p<0.05**  **sex within fl/fl: p<0.05**  sex within ΔOt: p>0.05 |
|  | miR21 - TA | Genotype 0.415  Sex 0.349  Genotype x Sex 0.082 | N/A |
