# Supplementary material for: miR21 deletion in osteocytes has direct and indirect effects on skeletal muscle in a sex-dimorphic manner in mice
Source: Biol Sex Differ. 2022 Oct 1;13:56. doi: 10.1186/s13293-022-00465-9 (PMC9526971; doi:10.1186/s13293-022-00465-9)
Supplement: Supplementary file 3 — Additional file 3: Fig. S1 Expression of miR21 in gastrocnemius and tibialis anterior (TA) muscles. Expression of miR21 corrected by miR135 levels in the gastrocnemius and TA muscles (N = 5–6/group) from female and male OtmiR21Δ and miR21fl/fl mice. Two-way ANOVA analyses were used to determine significant differences (Additional file 2: Table S2) * = p ≤ 0.05 compared to sex-matched miR21fl/fl controls, # = p < 0.05 for overall male versus female comparisons, and £ = p < 0.05 compared to females of the same genotype. [file 13293_2022_465_MOESM3_ESM.pptx]

## Slide 1
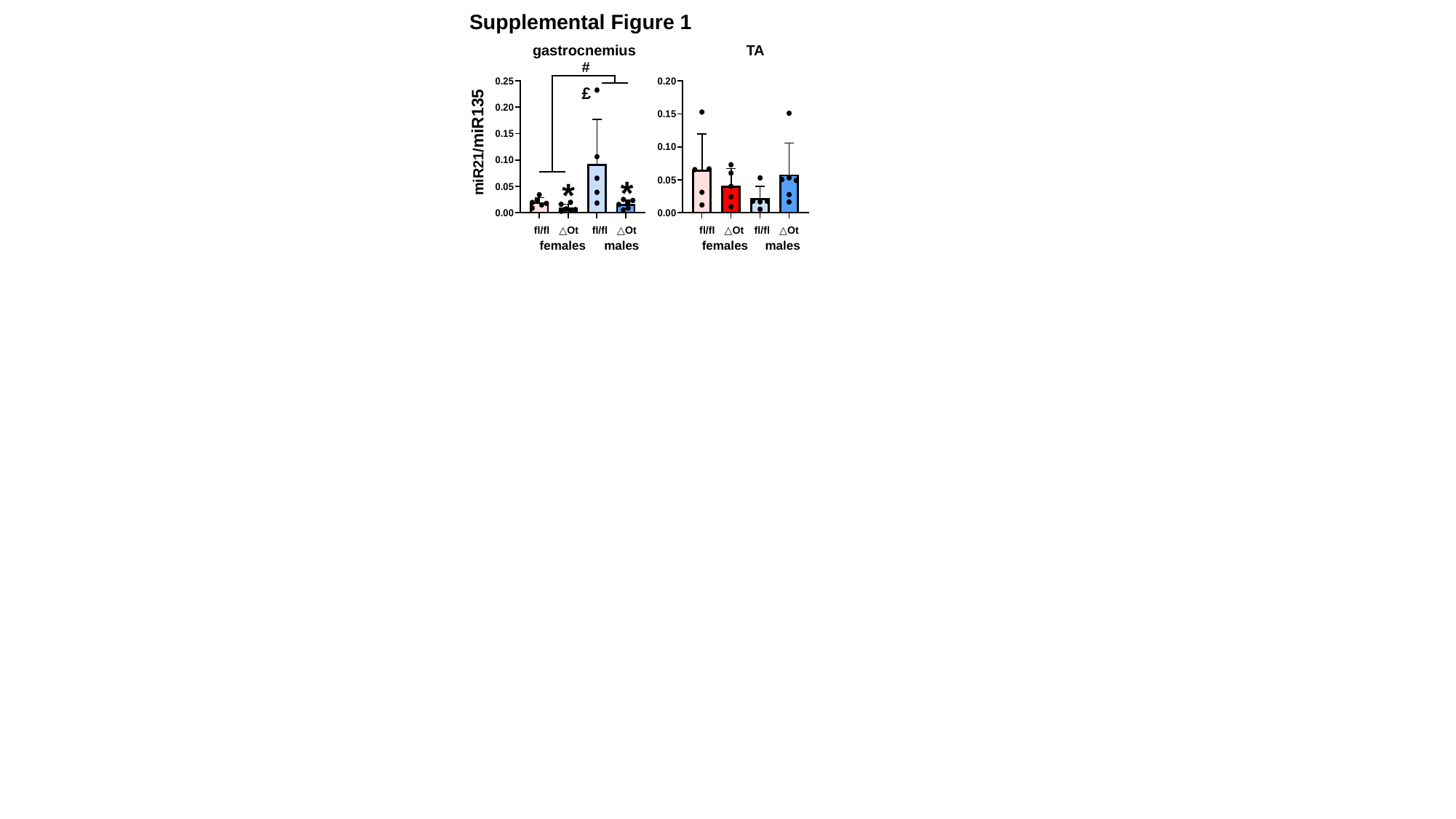

Supplemental Figure 1
gastrocnemius
TA
#
£
miR21/miR135
*
*
fl/fl
△Ot
fl/fl
△Ot
fl/fl
△Ot
fl/fl
△Ot
females
males
females
males
